# Evaluating the burden of head injuries on a rural emergency department in South Africa

**DOI:** 10.4102/safp.v63i1.5327

**Published:** 2021-10-25

**Authors:** Sannya Ramdheen, Bavani Naicker

**Affiliations:** 1 Division of Emergency Medicine, School of Clinical Medicine, University of KwaZulu-Natal, Durban, South Africa

**Keywords:** head injury, emergency department, trauma burden, low-middle income setting

## Abstract

**Background:**

Head injuries place a significant burden on the emergency department (ED) workload. This is prominent in low-middle income countries (LMICs), which have low resourced health systems and a skewed burden compared to global data. A large paucity of data exists among LMICs, therefore limiting comparisons on a global perspective. This study aimed to evaluate the ED burden of head injuries in a rural setting, within a LMIC.

**Methods:**

A retrospective chart review of all ED patients presenting with head injuries was conducted over a 3-month period. Relevant data was extracted using a data collection tool, followed by descriptive statistical analysis.

**Results:**

A total of 263 patients were identified, with a median age of 27 years and male predominance (78.7%). Interpersonal violence (IPV) was the mechanism of injury in 59.7% (*n* = 157) of cases, followed by road traffic injuries (23.2%) and non-intentional trauma (17.1%). Most injuries were because of blunt trauma (71.1%) and common types were soft tissue (46.2%) and scalp injuries (35.0%). In the paediatric subgroup, the most common mechanism of injury was falls, accounting for 52.0% of all falls in the study. The majority (71.5%) of patients were discharged, while 22.8% were admitted and 2.67% demised in the ED.

**Conclusion:**

At this rural centre, there is a high ED burden of minor head injuries because of IPV, with a strong male predominance. This study serves to add to limited reported data from a LMIC setting, which appears to have a skewed burden compared to the global data.

## Introduction

Traumatic brain injury (TBI) is a growing public health concern and represents the greatest contributor to death and disability globally amongst all trauma-related injuries.^1^ According to the World Health Organization (WHO), injuries account for 8% of global deaths, with road injuries exceeding interpersonal violence (IPV).^2^ Each year an estimated 69 million individuals will suffer a TBI, the vast majority of which will be mild (81.0%) and moderate (11.0%) in severity.^1^

The healthcare systems in low-middle income countries (LMICs) encounter nearly three times as many total TBIs than those in high income countries (HICs). These estimates are limited by relatively low-quality data from LMICs and suggest the need for more robust and accurate injury reporting.^1^ The Global Health Estimates report from 2019 showed that IPV, self-harm and road traffic injuries (RTI) rank fourth, eighth and ninth, respectively, in the top 10 causes of death amongst South African males of all age groups.^3^

The 2017 Statistics report of South Africa demonstrated that from 2006, the proportion of deaths attributed to non-natural causes in South Africa has steadily increased from 8.6% in 2006 to 11.5% in 2017. The population age groups largely affected by non-natural deaths are 15–19 years (43.1%), 20–24 years (47.9%) and 25–29 years (38.0%).^4^ Assault and transport accidents accounted for 26.5% of all non-natural deaths in 2017.^4^ KwaZulu-Natal (KZN) had the second highest (12.5%) proportion of deaths because of non-natural causes.

Head injury is a non-specific term, which includes clinically evident external injuries to the face, scalp, and calvarium, such as lacerations, contusions, abrasions, and fractures, and these may or may not be associated with TBI.^5,6^ The TBI is defined as a non-degenerative, non-congenital insult to the brain from an external mechanical force, possibly leading to permanent or temporary impairment of cognitive, physical and psychosocial functions, with an associated diminished or altered state of consciousness.^7^

The severity of head injuries may be assessed using several parameters including clinical examination, Glasgow Coma Scale (GCS) and other scoring systems. The Trauma Score (TS) has been revised to include GCS, systolic blood pressure, and respiratory rate. The Revised Trauma Score (RTS) yielded more accurate outcome predictions for patients with serious head injuries than the TS.^8^ A United Kingdom (UK) study evaluated the RTS compared to the Injury Severity Score (ISS). They recommend use of the RTS as an aid to junior doctors in early recognition of seriously injured patients in the emergency department (ED).^9^

Ngwelezana Hospital (NH) is a public sector facility based in rural KZN, South Africa. This 436 bedded hospital functions at district, regional and tertiary levels. It is located in the uMhlatuze sub-district and is the designated tertiary centre for King Cetshwayo, Zululand and Umkhanyakude districts. All patients are referred to NH via the Emergency Department (ED). A previous descriptive study at NH over a 5-year period showed a large trauma burden. Isolated head injury was the third highest mechanism of major trauma. The study highlighted the high incidences of both IPV and pedestrian-motor vehicle collisions (PMVCs) typical of trauma in a South African setting.^10^ A limitation of this previous study was that only patients classified as major trauma were included.

This study aimed to describe the ED burden and severity of head injuries in a resource limited, low-middle income setting. The objectives were to describe the spectrum of head injuries seen at a rural ED; to measure the contributions of IPV, road traffic incidents and non-intentional trauma; to find relationships between modes of injury and type of head injury sustained; and to evaluate severity of patients presenting with head injuries.

## Methods

A retrospective chart review was conducted at the facility for the period 01 October 2018 to 31 December 2018. The study included all patients who presented to the ED with a head injury during the consecutive 3-month period. Patients with secondary brain injury as a result of hypoxia, hypoglycaemia, post-cardiac arrest, hypotension, cerebral ischaemia and patients certified dead on arrival were excluded from the study.

The ED patient register was used to identify head injury patients who were treated within the study period. The charts were obtained either from the hospital records or mortuary. Using a data collection tool, relevant information was extracted and captured on Apple Numbers for statistical analysis. This included demographics, mechanism of injury, mode of injury, clinical signs, injury types, severity and disposition.

Descriptive analysis was performed by a statistician using Statistical Package for Social Sciences (SPSS) version 25. Pearson chi-squared test and Fisher’s exact test were used to test associations and a *p-*value less than 0.05 was considered statistically significant.

### Ethical considerations

Ethical approval was obtained from the Biomedical Research Ethics Committee (BREC) at University of KwaZulu-Natal (reference number: BE054/19). Hospital management at the study centre authorised access to patient records in line with BREC approval. Permission to conduct research was also obtained from the National Health Research Database (NHRD reference: KZ_201903_015). Patient confidentiality was maintained throughout the study.

## Results

**FIGURE 1:**
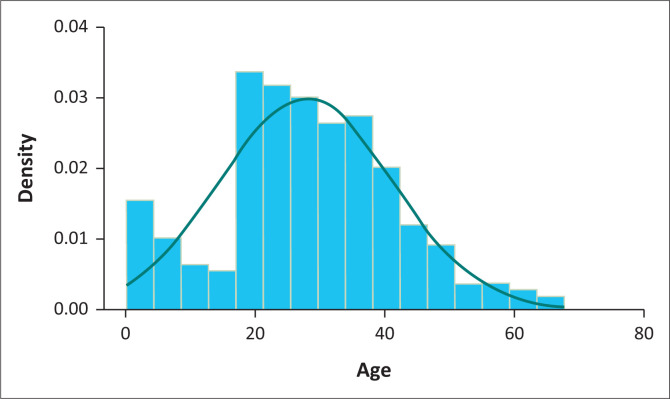
Patients’ age distribution (in years).

A total of 4502 patients were seen in the ED during the study period. Of these, 393 patients were identified with head injuries. This accounted for 8.7% of the total ED burden. However, only 263 patients were included as 95 records were not found and a further 35 had incomplete data or met exclusion criteria. The median age of the patients was 27 years with an interquartile range of 20–36 (Figure 1).

Males accounted for 78.7% of all cases (Table 1). Almost two-thirds (62.0%) of patients arrived using Emergency Medical Services (EMS). Blunt trauma was the mode of injury in 187 (71.1%) patients. Interpersonal violence was the mechanism of injury in 59.7% (*n* = 157) of the cases, followed by road traffic incidents (23.2%) and non-intentional trauma (17.1%). Males accounted for 87.0% (*n* = 136) of IPV cases. Within the RTI group, Motor Vehicle Accidents (MVA) accounted for 73.8% (*n* = 45), whilst Pedestrian Vehicle Accidents (PVA) accounted for 26.2% (*n* = 16). The majority of the study group (58.2%) were treated for isolated head injury, while the rest had concomitant injuries. Focal neurology was found in 17 patients (6.5%). In the paediatric subgroup, the most common mechanism of injury was falls, accounting for 52.0% of all falls in the study.

**TABLE 1:** Demographics according to age and gender distribution (*n* = 263).

Variable	Frequency		Age				Sex			
	
	*n*	%	Adult†		Paediatric‡		Male		Female	
			*n*	%	*n*	%	*n*	%	*n*	%
**Age**										
Adult†	232	88.2	-	-	-	-	186	80.2	46	19.8
Paediatric‡	31	11.8	-	-	-	-	21	67.7	10	32.3
Total	263	100.0	-	-	-	-	207	78.7	56	21.3
**Mode of transport**										
EMS	163	62.0	148	90.8	15	9.2	128	78.5	35	21.5
Private	100	38.0	84	84.0	16	16.0	79	79.0	21	21.0
**Mode of injury**										
Blunt	187	71.1	162	86.6	25	13.4	144	77.0	43	33.0
Penetrating	76	28.9	70	92.1	6	7.9	63	82.9	13	17.1
**Mechanism**										
IPV	157	59.7	156	99.4	1	0.6	136	86.6	21	13.4
RTI	61	23.2	55	90.2	6	9.8	42	68.9	19	31.1
MVA	45	73.8	41	91.1	4	8.9	27	60.0	18	40.0
PVA	16	26.2	14	87.5	2	12.5	15	93.8	1	6.2
Non-intentional	45	17.1	21	46.7	24	53.3	29	64.4	16	35.6
Fall	25	55.6	12	48.0	13	52.0	15	60.0	10	40.0
Other	20	44.4	9	45.0	11	55.0	14	70.0	6	30.0
**Isolated HI**	153	58.2	126	82.4	27	17.6	120	78.4	33	21.6
**Polytrauma**	110	41.8	106	96.4	4	3.6	87	79.1	23	20.9
**Focal neurology**	17	6.5	15	88.2	2	11.8	13	76.5	4	23.5
**No focal neurology**	246	93.5	217	88.2	29	11.8	194	78.9	52	21.1

EMS, emergency medical services; IPV, interpersonal violence; RTI, road traffic injuries; MVA, motor vehicle accident; PVA, pedestrian vehicle accident; HI, head injury.

† , Age 12 years and above.

‡ , Age below 12 years.

The most common types of injury were soft tissue, accounting for 46.2% of all injuries, followed by scalp injuries (35.0%) as demonstrated in Table 2. A proportion of patients (16.7%) had two or more types of injuries to the head. Soft tissue injuries, facial fractures, scalp injuries, skull fractures and intracranial injuries occurred more frequently because of blunt trauma compared to penetrating injuries. Road traffic injuries caused most (48.5%) of the intracranial injuries. However, all other types of injuries occurred most frequently because of IPV. Road traffic injuries and non-intentional trauma contributed equally to scalp injuries.

**TABLE 2:** Contributions of modes and mechanisms to different injury types (*n* = 263).

Injury type	Soft tissue (*f* = 165) (46.2%)		Facial fracture (*f* = 9) (2.5%)		Scalp injury (*f* = 125) (35.0%)		Skull fracture (*f* = 16) (4.5%)		Intracranial injury (*f* = 35) (9.8%)		Other (*f* = 7) (2.0%)	
	*f*	%	*f*	%	*f*	%	*f*	%	*f*	%	*f*	%
**Mode of injury**												
Blunt	122	73.9	5	55.6	85	68.0	9	56.2	29	82.9	4	57.1
Penetrating	43	26.1	4	44.4	40	32.0	7	43.8	6	17.1	3	42.9
**Mechanism**												
IPV	97	58.8	7	77.8	81	64.8	11	68.8	15	42.9	5	71.4
RTI	42	25.5	2	22.2	22	17.6	4	25.0	17	48.5	1	14.3
MVA	31	18.8	1	11.1	14	11.2	1	6.2	9	25.7	1	14.3
PVA	11	6.7	1	11.1	8	6.4	3	18.8	8	22.8	0	-
Non-intentional	26	15.7	0	-	22	17.6	1	6.2	3	8.6	1	14.3
Fall	12	7.3	0	-	14	11.2	1	6.2	1	2.9	0	-
Other	14	8.4	0	-	8	6.4	0	-	2	5.7	1	14.3

Note: Fourty four patients had two or more types of injuries.

*f*, frequency; IPV, interpersonal violence; RTI, road traffic injuries; MVA, motor vehicle accident; PVA, pedestrian vehicle accident.

Multiple scales were used to assess severity of head injuries (see Table 3). Severe head injury in adults was identified in 75 patients using triage early warning score (TEWS); 11 patients using GCS and three using RTS. Alert, Verbal, Pain, Unresponsive (AVPU) assessment was used for paediatric patients, which found two moderate and one severe injury. Overall, most of the injuries in the study were classified as mild.

**TABLE 3:** Severity of head injuries.

Severity	Scale			
	TEWS	AVPU‡	GCS†	RTS†
Mild	112	28	218	229
Moderate	76	2	3	-
Severe	75	1	11	3

TEWS, Triage Early Warning Score; AVPU, Alert, Verbal, Pain, Unresponsive; GCS, Glasgow Coma Scale; RTS, Revised Trauma Score.

† , GCS and RTS used to assess adults only (*n* = 232).

‡ , AVPU used to assess paediatric patients only (*n* = 31).

The majority (71.5%) of patients were discharged, while 22.8% were admitted and 2.7% (*n* = 7) demised in the ED (see Table 4). All deaths were adults with a total of five males (71.4%) and two females (28.6%). Of those requiring neurosurgery, 83.4% had an isolated head injury. There was concomitant injury in 60.0% of patients requiring admission and 71.4% of the deaths. Intensive care unit (ICU) admission was equally weighted (50.0%) with regard to gender, age group and isolated head injury versus concomitant injury.

**TABLE 4:** Disposition from emergency department (*n* = 263).

Variable	Disposition									
	Discharge (*f* = 188) (71.5%)		Admission (*f* = 60) (22.8%)		Neurosurgery (*f* = 6) (2.3%)		ICU (*f* = 2) (0.8%)		Demised (*f* = 7) (2.7%)	
	*f*	%	*f*	%	*f*	%	*f*	%	*f*	%
Adult (*n* = 232)	166	71.6	53	22.8	5	2.2	1	0.4	7	3.0
Paediatric (*n* = 31)	22	71.0	7	22.6	1	3.2	1	3.2	0	-
Male (*n* = 207)	143	69.1	52	25.1	6	2.9	1	0.5	5	2.4
Female (*n* = 56)	45	80.4	8	14.3	0	-	1	1.8	2	3.5
Isolated HI (*n* = 153)	121	79.1	24	15.6	5	3.3	1	0.7	2	1.3
Polytrauma (*n* = 110)	67	61.0	36	32.7	1	0.9	1	0.9	5	4.5

*f*, frequency; ICU, intensive care unit; HI, head Injury.

## Discussion

The purpose of this study was to demonstrate the burden of head injuries in a rural ED, within a LMIC setting. It shows a strong male predominance, with a 3.7:1 male to female ratio and the highest burden from the 20 to 36 year age group. Interpersonal violence was the most frequent mechanism of injury for both genders. A similar study done in Botswana demonstrated a significant burden of head trauma presenting to their largest ED. There were over twice as many males as females, and 80% of head injury patients were aged below 40 years, which is similar to previously published sub-Saharan Africa figures. However, the aetiology was equally attributable to RTI and IPV in that study.^6^ Global data indicates that majority (60.0%) of TBIs are because of RTIs; 20% – 30% as a result falls; 10% from IPV, and 10% because of work and sport related injuries.^11^ This study demonstrates a skewed burden comparatively.

Almost 9% of all the patients seen at this ED during the study period presented with head injuries. This demonstrates an alarming burden and emphasises the need for improvement of national public health to alleviate this burden on our low resourced ED’s. Tying this with the key findings of IPV, male predominance and a 20–36 year age group, this should be our targeted group for further investigation within this population to identify key causative factors and preventative measures such as socio-economic background, education and employment status. Another possible key contributor which hasn’t been included in this study is the relationship with alcohol and these injuries.

The paediatric group was defined as age below 12 years. More children were transported to hospital using private vehicles compared to the use of EMS. Perhaps this is because of overwhelmed EMS services or a lack of availability thereof, forcing caregivers to seek alternate means of transport. The predominant aetiology was non-intentional trauma with falls as the major mechanism. Bruns et al. found that falls predominated as a cause of injury in the children and elderly, regardless of race and gender in their global review of epidemiology of TBI.^5^ Other non-intentional mechanisms of injury observed in our study were kicks from animals, sport injuries and solid objects accidentally striking the head such as trees, gates and bricks. As most of the paediatric injuries were non-intentional, it will be difficult to find impactful preventative measures for this group.

Interpersonal violence was found in one child. Isolated head injury was seen in 87.1% of paediatric patients with most cases being mild in severity (90.0%) and 71.0% discharged home. This was congruent with a global qualitative review of paediatric TBI by Dewan et al., which showed mild TBI in more than 80.0% of children, with falls and motor vehicle collision representing the major mechanisms of injury.^12^ There were no paediatric mortalities observed in this study.

Our study demonstrated that there was a higher incidence of blunt trauma compared to penetrating injuries in the head injured population. The most common type of injuries were soft tissue, which included facial swelling, abrasion or laceration. Interpersonal violence was the most common cause of these soft tissue injuries. Some of the objects used for blunt assault in the IPV group included fists, sticks, unbroken bottles and solid objects. Penetrating IPV related injuries were caused by assault with bush knives, stabbing with broken bottles or knives and gunshot wounds. Scalp injury was defined as a haematoma, abrasion or laceration to any region of the scalp. The spectrum of intracranial injuries observed in the study group was intracranial haemorrhage, contusions, diffuse axonal injury, and depressed skull fractures. Most of the intracranial injuries observed were because of RTI. Other injuries to the head included ear lacerations, blunt or penetrating eye trauma and a palate injury.

Road traffic injuries refer to injuries sustained by motorists, cyclists or pedestrians. A scarcity of sidewalks and traffic lights, in addition to poor helmet compliance in LMICs is thought to contribute to a higher rate of head injuries following RTI.^1^ Furthermore, inadequate safety technology or overcapacity of vehicles can compound an otherwise trivial collision. For example, a single collision involving a truck full of unrestrained occupants can result in a mass casualty incident with multiple TBI cases – a scenario not frequently observed in HICs.^1^

Triage early warning score is performed upon first contact when patients arrive at the ED. It incorporates vital signs, level of consciousness according to AVPU, mobility of the patient and presence of trauma to assess patient severity. The TEWS predicts in-hospital mortality within 24 h, 48 h, 7 days and 30 days better than the National Early Warning Score (NEWS), Modified Early Warning Score (MEWS) and Rapid Emergency Medicine Score (REMS) for patients arriving at the ED.^13^

The AVPU is a straightforward scale that is useful to rapidly grade a patient’s gross level of consciousness, responsiveness or mental status.^14^ The specific criteria and severity interpretations are: alert (mild), verbally responsive (moderate), painfully responsive (moderate) or unresponsive (severe).

The GCS was first published in 1974 at the University of Glasgow by neurosurgery professors Graham Teasdale and Bryan Jennett. The GCS is used to objectively describe the extent of impaired consciousness in all types of acute medical and trauma patients. The scale assesses patients according to three aspects of responsiveness: eye-opening, motor and verbal responses.^15,16^ The maximum score is 15 and its universally subdivided into mild (13–15), moderate (9–12) and severe (3–8) groups. One of the expressed reservations regarding the GCS has been its failure to incorporate brainstem reflexes. The scale also includes a numerical skew towards the motor response. Despite its drawbacks, the GCS remains the most universally utilised level of consciousness scale worldwide.^16^ The GCS was assessed in the adult sub-group and found that 94.0% were mild, 1.3% moderate and 4.7% severe head injuries. This is comparative to the South African epidemiology study which showed 88.0% mild, 8.0% moderate and 5% severe.^5^

Access to neurosurgery is limited in a rural setting. Patients from the study centre requiring intervention have to be transported approximately 174 km away by EMS. There are further constraints as the referral centre has a large drainage area and often does not have the bed capacity required to accept more patients. Therefore, while the number of patients referred to neurosurgery appears low in this study, it may not be a true reflection of the actual number of patients that may have needed intervention, but did not make it on time. No follow up was done after disposition from the ED.

## Limitations

Some of the limitations that were identified during the study were poor record keeping. Almost 25% of records were not found. In addition, some records that were analysed had missing data, resulting in further exclusion. The accuracy of data presented relies on good record keeping by attending personnel. This was a single centred study and may not account for the minor cases that were treated and discharged from primary level services within the same district. Patients who were dead on arrival were excluded. No follow up was done after disposition from the ED, therefore patients that demised outside the ED setting are not accounted for. These two factors may result in under-reporting of severe head injuries. The study focused on describing data from a rural LMIC setting and did not include urban areas within the LMIC.

## Conclusion

At this rural centre, there is a high ED burden of minor head injuries because of IPV, with a strong male predominance. Although this study may not directly change clinical practice, it serves to add to limited reported data from a LMIC setting, which appears to have a skewed burden compared to global data. The demographics and injury patterns found in this study may be used to drive future public health interventions to address this growing burden of head injuries. Further studies are necessary to add to this data, with the end goal to improve quality of care of ED; improve outcomes; and to develop prevention strategies to reduce the overall burden.
